# Genetic Variants That Confer Resistance to Malaria Are Associated with Red Blood Cell Traits in African-Americans: An Electronic Medical Record-based Genome-Wide Association Study

**DOI:** 10.1534/g3.113.006452

**Published:** 2013-07-01

**Authors:** Keyue Ding, Mariza de Andrade, Teri A. Manolio, Dana C. Crawford, Laura J. Rasmussen-Torvik, Marylyn D. Ritchie, Joshua C. Denny, Daniel R. Masys, Hayan Jouni, Jennifer A. Pachecho, Abel N. Kho, Dan M. Roden, Rex Chisholm, Iftikhar J. Kullo

**Affiliations:** * Division of Cardiovascular Diseases, Mayo Clinic, Rochester, Minnesota 55905; *** Department of Internal Medicine, Mayo Clinic, Rochester Minnesota 55905; † Division of Biomedical Statistics and Informatics, Mayo Clinic, Rochester, Minnesota 55905; ‡ Office of Population Genomics, National Human Genome Research Institute (NHGRI), Bethesda, Maryland 20892; ‡‡ Departments of Biomedical Informatics, Medicine and Pharmacology, Vanderbilt University School of Medicine, Nashville, Tennessee 37232; § Center for Human Genetics Research, Vanderbilt University, Nashville, Tennessee 37232; †† Department of Biochemistry and Molecular Biology, The Pennsylvania State University, University Park, Pennsylvania 16802; §§ Department of Biomedical Informatics and Medical Education, University of Washington, Seattle, Washington 98195; ‡‡‡ Center for Genetic Medicine, Northwestern University Feinberg School of Medicine, Chicago Illinois 60611; ** Department of Preventive Medicine, Northwestern University Feinberg School of Medicine, Chicago, Illinois 60611; ††† Departments of Medicine and Preventive Medicine, Northwestern University, Chicago, Illinois 60611

**Keywords:** red blood cell (RBC) traits, genome-wide association study, African-Americans, natural selection, informatics, electronic medical record

## Abstract

To identify novel genetic loci influencing interindividual variation in red blood cell (RBC) traits in African-Americans, we conducted a genome-wide association study (GWAS) in 2315 individuals, divided into discovery (*n* = 1904) and replication (*n* = 411) cohorts. The traits included hemoglobin concentration (HGB), hematocrit (HCT), RBC count, mean corpuscular volume (MCV), mean corpuscular hemoglobin (MCH), and mean corpuscular hemoglobin concentration (MCHC). Patients were participants in the electronic MEdical Records and GEnomics (eMERGE) network and underwent genotyping of ~1.2 million single-nucleotide polymorphisms on the Illumina Human1M-Duo array. Association analyses were performed adjusting for age, sex, site, and population stratification. Three loci previously associated with resistance to malaria—*HBB* (11p15.4), *HBA1/HBA2* (16p13.3), and *G6PD* (Xq28)—were associated (*P* ≤ 1 × 10^−6^) with RBC traits in the discovery cohort. The loci replicated in the replication cohort (*P* ≤ 0.02), and were significant at a genome-wide significance level (*P* < 5 × 10^−8^) in the combined cohort. The proportions of variance in RBC traits explained by significant variants at these loci were as follows: rs7120391 (near *HBB*) 1.3% of MCHC, rs9924561 (near *HBA1/A2*) 5.5% of MCV, 6.9% of MCH and 2.9% of MCHC, and rs1050828 (in *G6PD*) 2.4% of RBC count, 2.9% of MCV, and 1.4% of MCH, respectively. We were not able to replicate loci identified by a previous GWAS of RBC traits in a European ancestry cohort of similar sample size, suggesting that the genetic architecture of RBC traits differs by race. In conclusion, genetic variants that confer resistance to malaria are associated with RBC traits in African-Americans.

Disorders involving red blood cells (RBCs) are common and associated with adverse health outcomes (de Simone *et al.* 2005; Letcher *et al.* 1983; Sarnak *et al.* 2002; Sharp *et al.* 1996). Such disorders, including iron deficiency anemia, sickle-cell disease, and glucose-6-phosphate dehydrogenase (G6PD) deficiency, affect millions of people around the world and are a major cause of morbidity and mortality. RBC traits, including hemoglobin concentration (HGB), hematocrit (HCT), RBC count, mean corpuscular volume (MCV), mean corpuscular hemoglobin (MCH), and mean corpuscular hemoglobin concentration (MCHC), are commonly measured as part of the complete blood count. The RBC traits have a substantial genetic component, with heritabilities of 0.56, 0.52, and 0.52 reported for RBC count, MCV, and MCH, respectively (Lin *et al.* 2007).

Genome-wide association studies (GWAS) have revealed multiple loci that influence interindividual variation in RBC traits in individuals of European and Asian ancestry (Ding *et al.* 2012; Ganesh *et al.* 2009; Kamatani *et al.* 2010; Kullo *et al.* 2010; Soranzo *et al.* 2009). Whether these and additional novel loci influence RBC traits in individuals of recent African ancestry is unknown. A GWAS for RBC traits in patients of recent African ancestry (*i.e.*, African Americans) has yet to be reported. Identifying common genetic variants influencing RBC traits may offer insights into iron metabolism and erythropoiesis in African Americans, and such variants may also modify disease severity in conditions such as sickle-cell disease and thalassemia. It is well known for example, that sickle-cell heterozygotes have less severe incidences of *Plasmodium falciparum* infections than those with normal adult hemoglobin. Other abnormal hemoglobins, G6PD deficiency and pyruvate kinase deficiency also confer some degree of resistance against falciparum malaria (reviewed by Allison 2009).

Differences in RBC traits between African Americans and non-Hispanic white subjects have been observed in previous studies (Beutler and West 2005; Perry *et al.* 1992). Compared with non-Hispanic white individuals, African Americans have lower hemoglobin levels, lower hematocrit levels, lower MCV, lower serum transferrin saturation, and greater levels of serum ferritin (Beutler and West 2005). Such differences have been attributed to socioeconomic, nutritional (Jackson 1990) and genetic factors (Perry *et al.* 1992). To identify novel genetic loci influencing interindividual variation in RBC traits, we conducted a two-stage GWAS in 2315 African-American patients participating in the electronic MEdical Records and GEnomics (eMERGE) network. The network (www.gwas.org) was established by the National Human Genome Research Institute to develop and implement approaches for leveraging biorepositories associated with EMR systems for large-scale genomic research (Kho *et al.* 2011; Manolio 2009; McCarty *et al.* 2011). We have previously reported results of a GWAS for RBC traits in 3012 patients of European ancestry in the Mayo eMERGE cohort (Kullo *et al.* 2011), and in 12,486 patients of European ancestry from the entire eMERGE network (Ding *et al.* 2012). We investigated whether loci identified in these and other cohorts of European and Asian ancestry were associated with RBC traits in African-Americans.

## Methods

### Study sample

A total of 1904 eMERGE phase I African-American patients in the Vanderbilt University Medical Center and Northwestern University biorepositories served as the discovery cohort. These individuals were selected and genotyped for a quantitative trait analysis of normal cardiac conduction led by Vanderbilt University Medical Center (Denny *et al.* 2010) and a case-control study of type II diabetes led by Northwestern University (Kho *et al.* 2012), respectively. An additional 411 patients of African-American ancestry, enrolled at these sites for a GWAS of resistant hypertension, served as the replication cohort.

### Genotyping and quality control

Genotyping was performed on the Illumina Human 1M-Duo platform at the Broad Institute of Harvard and Massachusetts Institute of Technology and for additional samples used for the resistant hypertension GWAS, at the Center for Inherited Disease Research at Johns Hopkins University. The platform includes ~1.2 million markers with a median spacing between markers of 1.5 kb (mean = 2.4 kb). These markers provide 76% coverage of the genome of an African population at *r*^2^ > 0.8 (www.illumina.com/Documents/products/datasheets/datasheet_infiniumhd.pdf). Genotype data were cleaned using the quality control (QC) pipeline developed by the eMERGE Genomics Working Group (Turner *et al.* 2011; Zuvich *et al.* 2011). The process includes evaluation of sample and marker call rate, gender mismatch and anomalies, duplicate and HapMap concordance, batch effects, Hardy-Weinberg equilibrium, sample relatedness, and population stratification. A total of 907,954 single-nucleotide polymorphisms (SNPs) in the Illumina 1M-Duo array were available for analysis after we applied the following QC criteria: SNP call rate >98%, sample call rate >98%, minor allele frequency >0.01, Hardy-Weinberg equilibrium *P* >0.001, and 99.99% concordance rate in duplicates. One sample each from any related pairs was removed. After QC, 2315 patients African-Americans with phenotype and genotype data were available for association analyses.

### Statistical analysis

When multiple measurements of a RBC trait were available for an individual patient, we chose the median value and the corresponding age for the genetic analyses. We performed association analyses by using linear regression implemented in PLINK (Purcell *et al.* 2007), assuming additive genetic effects, with adjustment for age, sex, site, and for any population substructure (*i.e.*, the first two principal components [PCs]). We adjusted for genetic ancestry via the first two PCs generated by principal component analysis (PCA), a mathematical procedure that uses an orthogonal transformation to convert a set of observations of possibly correlated variables (n × m matrix, *i.e.*, sample × genotypes matrix) into a set of values of linearly uncorrelated variables called PCs. For SNPs in the X chromosome, alleles A and B were coded (A → 0; and B → 1) in males and (AA → 0; AB → 1, and BB → 2) in females, and additionally sex was included as a covariate. We estimated the regression coefficient (*β*), and the proportion of variance in a RBC trait explained by a variant (*i.e.*, *R*^2^). The statistical power for a sample size of 1904 in the discovery cohort to detect a quantitative trait locus that explained ~2% variance in a RBC trait, was 80% at a significance level of 5×10^−8^.

Given the known correlation between RBC traits, we performed PCA to identify the main vectors along which the RBC traits lie. These vectors were then used as phenotypes in the association analyses, with adjustments being the same as for the single SNP analyses.

Because RBC traits can be affected by a wide array of medical conditions, we also performed analyses in the subset of patients (*n* = 2005) in whom relevant comorbid conditions, specific medications, and blood loss were absent. To do this, we employed a previously developed algorithm based on billing codes and natural language processing of unstructured clinical notes to exclude RBC traits values affected by comorbidities, medications, or blood loss (Ding *et al.* 2012; Kullo *et al.* 2010, 2011). The phenotyping algorithm is available online (www.gwas.org), and the values of RBC traits before and after implementing the algorithm are provided in Supporting Information, Table S1).

Patterns of linkage disequilibrium (LD) were analyzed based on HapMap Phase II YRI for chromosome X (The International Hapmap Consortium 2005) and the 1000 Genome YRI for autosomal chromosomes (Durbin *et al.* 2010) via the LocusZoom software (Pruim *et al.* 2010). Map and pedigree files were not available for chromosome X in the 1000 Genome YRI in the LocusZoom package.

### Replication of significant loci identified in individuals of European ancestry

We selected the most significant SNPs in 15 loci associated with six RBC traits (HGB, HCT, RBC count, MCV, MCH, and MCHC) in individuals of European ancestry (Ding *et al.* 2012), and tested their associations in the study cohort.

## Results

The characteristics of study patients, including the discovery and replication cohorts, are summarized in Table 1. The number of individuals with RBC traits measured was as follows: 2244 for HGB, RBC count, MCV, MCH, and MCHC; and 2312 for HCT. The correlation matrix between the six RBC traits is shown in Table S2. The strongest correlation was between HGB and HCT (*r* = 0.95), and the weakest was between MCHC and RBC count (*r* = −0.03).

**Table 1:** Sample characteristics

	Discovery Cohort (*n* = 1904)		Replication Cohort (*n* = 411)	
	VUMC (*n* = 1561)	NU (*n* = 343)	VUMC (*n* = 276)	NU (*n* = 135)
Women, *n* (%)	1048 (67%)	250 (73%)	193 (70%)	100 (74%)
Age,*^a^* years	46.73 ± 16.31	49.85 ± 13.37	56.82 ± 13.68	41.98 ± 13.27
HGB, g/dL	12.67 ± 1.57	12.30 ± 1.54	12.57 ± 1.62	12.46 ± 1.66
HCT, %	38.71 ± 4.25	36.58 ± 4.49	38.41 ± 4.49	37.0 ± 4.91
RBC count, ×10^12^/L	4.51 ± 0.54	4.28 ± 0.59	4.43 ± 0.61	4.33 ± 0.63
MCV, fL	86.24 ± 6.39	86.22 ± 6.19	87.36 ± 6.53	86.37 ± 6.17
MCH, pg	28.22 ± 2.49	28.99 ± 2.38	28.56 ± 2.6	29.12 ± 2.41
MCHC, % or g/dL	32.70 ± 1.23	33.60 ± 0.68	32.67 ± 1.17	33.68 ± 0.75

VUMC, Vanderbilt University Medical Center; NU, Northwestern University; HGB, hemoglobin; HCT, hematocrit; RBC, red blood cell, MCV, mean corpuscular volume; MCH, mean corpuscular hemoglobin; MCHC, mean corpuscular hemoglobin concentration.

a The age is the age for median HGB. For the remaining traits, the age was within ±1 yr of the age at median HGB. In the association analyses for each trait, we used the corresponding median age for an individual. All trait values are mean ± SD.

**Figure 1:**
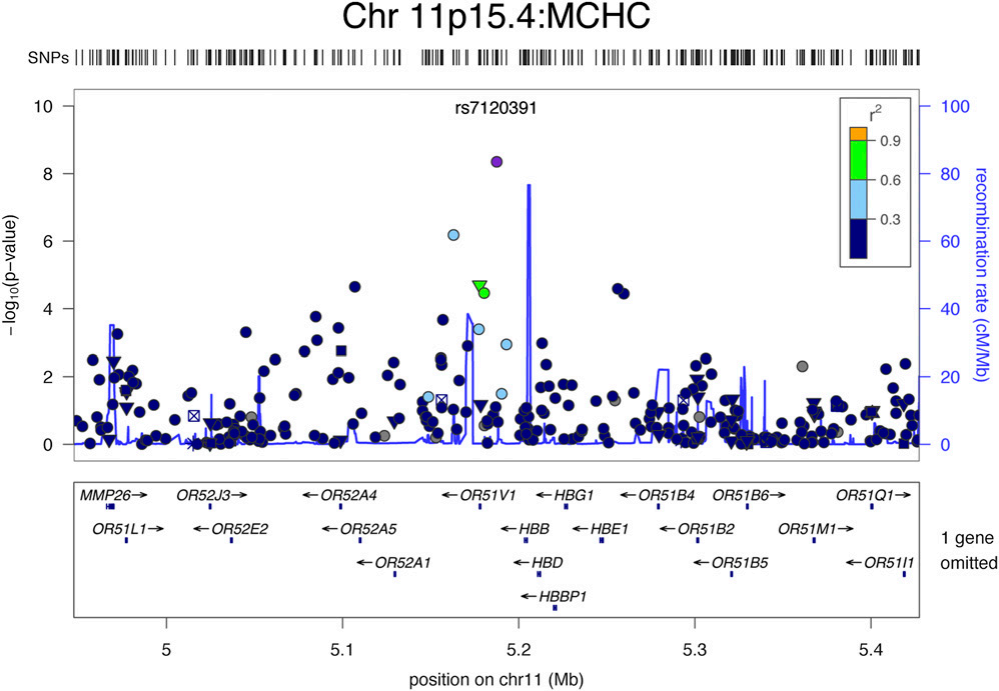
Regional plot of SNPs on chromosome 11p15.4 associated with MCHC. SNPs are plotted by position on the chromosome (x-axis) *vs.* (–log10 *P*) on y-axis. rs number for the most significant SNP is shown on the plot. Estimated recombination rates (from HapMap) are plotted in cyan to reflect the local LD structure. The SNPs near the most significant SNP are color coded to reflect their LD with this SNP (taken from pairwise *r*^2^ values from the HapMap CEU data). Genes, the position of exons and the direction of transcription from the UCSC genome browser are also plotted. ▼: non-synonymous; ●: no annotation; ☒:conserved in mammals; *: TFBS conserved

In the discovery cohort, we identified 51 SNPs associated with at least one of the six RBC traits at *P* < 1 × 10^−6^. We selected the 12 most significant SNPs based on the LD pattern (one for HGB; three each for RBC, MCV, and MCH; and two for MCHC; these 12 SNPs are independent; *i.e.*, *r*^2^ < 0.3) associated with six RBC traits in the discovery cohort, and tested their association in the replication cohort. After adjustment for multiple testing by Bonferroni correction (*i.e.*, the threshold for significance was 0.05/the number of tested SNPs for each trait), we identified three loci that were significant in the replication cohort and remained significant (*P* < 5 × 10^−8^) in the combined cohort: chromosome 11p15.4 (with MCHC), 16p13.3 (with MCV, MCH, and MCHC), and chromosome Xq28 (with RBC count, MCV, and MCH) (Figure 1) (Table 2). Boxplots of the distribution of RBC traits for the genotypes of the three significant SNPs are shown in Figure S1. We summarize below our results by chromosomal region.

**Table 2:** Genetic variants associated with RBC traits in African Americans

						Discovery Cohort (*n* = 1904)			Replication Cohort (*n* = 411)			Combined Cohort (*n* = 2315)		
Trait	Chr	SNP	Position*^a^*	Alleles*^b^*	Gene	MAF	*β* ± SE*^c^*	*P*	MAF	*β* ± SE	*P*	MAF	*β* ± SE	*P*
MCHC	11p15.4	rs7120391	5187483	C/T	*NA*	0.12	0.29 ± 0.06	3E-07	0.11	0.33 ± 0.12	5E-03	0.12	0.30 ± 0.05	5E-09
MCV	16p13.3	rs9924561	254781	T/G	*ITFG3*	0.09	−3.34 ± 0.36	2E-20	0.11	−4.63 ± 0.68	3E-11	0.10	−3.57 ± 0.32	5E-29
MCH	16p13.3	rs9924561	254781	T/G	*ITFG3*	0.09	−1.46 ± 0.14	2E-25	0.11	−1.99 ± 0.27	5E-13	0.10	−1.56 ± 0.12	8E-36
MCHC	16p13.3	rs9924561	254781	T/G	*ITFG3*	0.09	−0.44 ± 0.06	4E-12	0.12	−0.56 ± 0.11	1E-06	0.10	−0.47 ± 0.06	9E-17
RBC count	Xq28	rs1050828	153417411	A/G	*G6PD*	0.12	−0.20 ± 0.03	3E-12	0.12	−0.17 ± 0.07	0.02	0.12	−0.20 ± 0.03	4E-13
MCV	Xq28	rs1050828	153417411	A/G	*G6PD*	0.12	2.43 ± 0.35	4E-12	0.12	2.59 ± 0.78	9E-04	0.12	2.46 ± 0.32	1E-14
MCH	Xq28	rs1050828	153417411	A/G	*G6PD*	0.12	0.71 ± 0.14	2E-07	0.12	0.76 ± 0.31	0.01	0.12	0.72 ± 0.12	9E-09

RBC, red blood cell; Chr, chromosome; SNP, single-nucleotide polymorphism; MAF, minor allele frequency; MCHC, mean corpuscular hemoglobin concentration; MCV, mean corpuscular volume; MCH, mean corpuscular hemoglobin; NCBI, National Center for Biotechnology Information.

a NCBI human reference genome build 36.

b The first allele is the minor allele.

c *β* = regression coefficient; for the additive effects of SNPs, the direction of the regression coefficient represents the effect of each extra minor allele.

### Chromosome 11p15.4

An intergenic SNP (rs7120391) on chromosome 11p15.4, located 14.5 kb downstream of the *β*-hemoglobin gene (*HBB*, OMIM 141900), was associated with MCHC in the combined cohort (β = 0.30, *P* = 5 × 10^−9^; *R*^2^ = 1.3%; Figure 1).

### Chromosome 16p13.3

**Figure 2:**
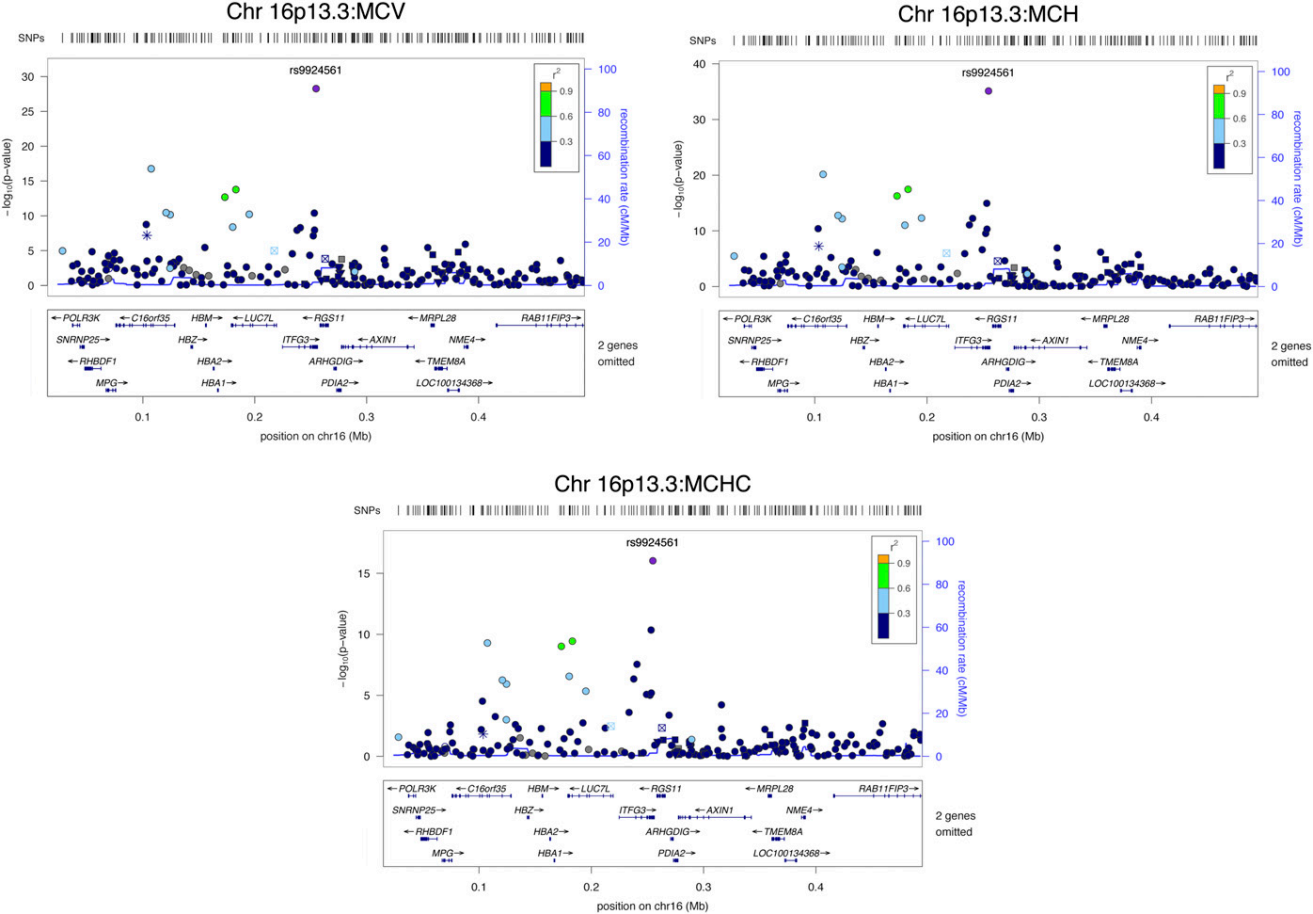
Regional plots of SNPs on chromosomes 16p13.3 associated with MCV, MCH, and MCHC.

A SNP (rs9924561) in an intron of the integrin α FG-GAP repeat containing 3 gene (*ITFG3*) was associated with MCV (β = −3.57, *P* = 5 × 10^−29^; *R*^2^ = 5.5%), MCH (β = −1.56, *P* = 8 × 10^−36^; *R*^2^ = 6.9%), and MCHC (β = −0.47, *P* = 9 × 10^−17^; *R*^2^ = 2.9%) in the combined cohort (Figure 2). Of note, the human α-globin gene cluster (*HBZ-HBM-HBA2-HBA1-HBQ1*), which contains three functional genes (ζ-, α2-, and α1-globin), is present at this locus (*HBA1* is ~87.2 kB upstream of rs9924561). Another SNP (rs13336641) adjacent to the α-globin gene cluster was associated with MCV (*P* = 2 × 10^−17^; *R*^2^ = 3.1%) and MCH (*P* = 7 × 10^−21^; *R*^2^ = 3.8%). The SNPs rs13336641 and rs9924561, although ~147 kb apart, are in LD (*r*^2^ = 0.46 in 1000 genomes YRI).

### Chromosome Xq28

**Figure 3:**
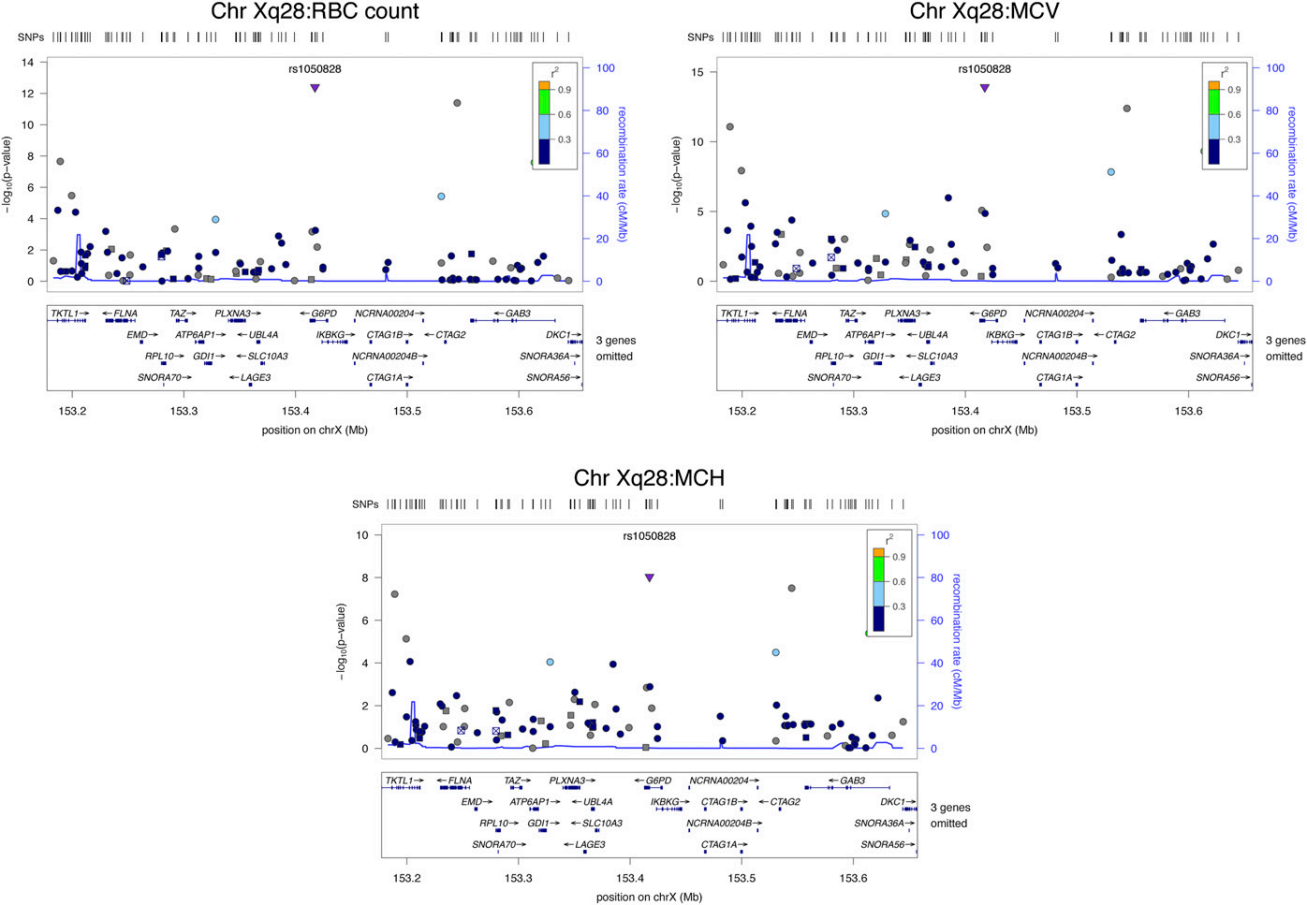
Regional plots of SNPs on chromosome Xq28 associated with RBC count, MCV, and MCH.

A nonsynonymous SNP (rs1050828, V→M) in the *G6PD* gene was associated with RBC count (β = −0.20, *P* = 4 × 10^−13^; *R*^2^ = 2.4%), MCV (β = 2.46, *P* = 1 × 10^−14^; *R*^2^ = 2.9%), and MCH (β = 0.72, *P* = 9 × 10^−9^; *R*^2^ = 1.4%) in the combined cohort (Figure 3). The minor allele (A) was associated with a lower RBC count and greater MCV and MCH.

We repeated our analyses after excluding 310 patients affected by various medical conditions (*i.e.*, hematologic disorders, medications, and blood loss) that can affect RBC traits, based on a previously developed algorithm (Kullo *et al.* 2010, 2011). In the subset of 2005 remaining patients, variants in the two of three chromosome loci (16p13.3 and Xq28) remained significant at a genome-wide significance level although significance levels decreased likely due to the smaller sample size (analyses not shown), although it is also possible that the medical conditions in some way biased the original association.

### Principal component analyses

The first three PCs explained 99% of the total variance among the correlated RBC traits. For the genomic association analyses, we used only the first and second PC (that explained 86% of the total variance, see Figure S2). The traits HCT, HGB, RBC count and MCH, MCV, RBC count were the main contributors to PC1 and PC2, respectively. The main driver of the third PC was MCHC (loading of −0.84, explained 13% of the total variance), which is similar to the genome-wide association analysis of this particular trait.

Two SNPs on chromosome 16p (*P* < 5 × 10^−8^) including rs9924561 that was identified for RBC count, HGB and HCT traits separately, were associated with PC1 (Table 3 and Figure S3). Nineteen SNPs either on chromosome 16p or Xq23 (*P* < 5 × 10^−8^) were associated with PC2 including rs9924561 and rs1050828 previously identified for MCV, MCH, and MCHC traits separately (Table 3 and Figure S4). The finding on chromosome 11 was not replicated in the PCAs.

**Table 3:** Genetic variants associated with the two PCs representing majority of variation in RBC traits

Vector*^a^*	CHR	SNP	Alleles*^b^*	Gene	*β* ± SE*^c^*	*P*
PC1	16p13.3	rs9924561	T/G	*ITFG3*	1.53 ± 0.21	4E-13
PC2	16p13.3	rs9924561	T/G	*ITFG3*	2.97 ± 0.25	1E-31
PC2	Xq28	rs1050828	A/G	*G6PD*	−1.97 ± 0.25	6E-15

PCs, principal components; CHR, chromosome; SNP, single-nucleotide polymorphism; HCT, hematocrit; HGB, hemoglobin; RBC, red blood cell; MCHC, MCV, mean corpuscular volume; MCH, mean corpuscular hemoglobin.

a PC1: HCT, HGB, and RBC count; PC2: MCV, MCH, and RBC count.

b The first allele is the minor allele.

c *β* = regression coefficient; for the additive effects of SNPs, the direction of the regression coefficient represents the effect of each extra minor allele.

### Replication of significant loci identified in individuals of European ancestry

SNPs at two loci [rs218237 on chromosome 4q12 [associated with RBC (*P* = 7 × 10^−4^), MCV (*P* = 1 × 10^−3^), and MCH (*P* = 3 × 10^−3^)] and rs7775698 on chromosome 6q23.3 [associated with RBC (*P* = 1 × 10^−2^) and MCH (*P* = 1 × 10^−3^)] were replicated in our study sample after correction for multiple comparisons. At both loci, the minor allele frequency, and the direction of association were similar between patients of European and African ancestry. The inability to replicate other loci may have been due low statistical power given the relatively small sample size of 2315 African Americans (*e.g.*, for 14 of 18 tests at the remaining 13 loci, our power to detect an association was < 80%); and/or a different genetic architecture underlying RBC traits in the two ethnic groups.

## Discussion

In the present study, using a two-stage design, we conducted a GWAS of 2319 African-American patients to identify novel genetic loci influencing interindividual variation in RBC traits. Three loci –11p15.4, 16p13.3, and Xq28-were associated with RBC traits. Variants in genes residing at these three loci (*HBB*, *HBA1*/*HBA2*, and *G6PD*) confer resistance to malaria in Africans (Kwiatkowski 2005). Malaria is a powerful force of natural selection that has shaped the pattern of variation in the human genome and led to the persistence of thalassemia, sickle-cell disease, and G6PD deficiency (Kwiatkowski 2005), with the latter two diseases occurring almost exclusively in people of recent African ancestry. Variants in at least nine genes related to erythrocyte structure and function have been associated with resistance to malaria (reviewed by Kwiatkowski 2005), including *HBB*, *HBA*, and *G6PD*. Our results indicate that variants in malaria-resistance genes influence RBC traits in individuals of recent African ancestry.

It is estimated that 1 in 12 African-American individuals in the United States has the sickle-cell trait. *HBB* is one of the classical examples of genes under natural selection. HbS homozygotes suffer sickle-cell disease, but heterozygotes have a 10-fold reduced risk of severe malaria. SNP rs7120391 downstream of the hemoglobin β gene (HBB) at chromosome 11p15.4 was associated with MCHC in the present study. The HbS allele (Glu6Val in HBB, rs334) leading to sickle-cell anemia, is in LD with rs7120391 (r^2^ = 0.37). MCHC is thought to be important in sickle-cell disease because sickling rates are strongly dependent on hemoglobin concentration (Clark *et al.* 1982).

Variants at chromosome 16p13.3 locus have also been reported to be associated with RBC traits in a GWAS of European ancestry individuals (Ganesh *et al.* 2009), and a candidate-gene based association study in 7112 African Americans (Lo *et al.* 2011). LD analysis indicated that the most significant SNP (rs9924561) in *ITFG3* is in high LD with significant SNPs (*e.g.*, rs13336641) in the 5′ regulatory region of the human α-globin gene cluster, including two *HBA* genes (*HBA1* and *HBA2*). Variants in *HBA* leading to α+ thalassemia are protective against severe malaria (Williams *et al.* 2005). Therefore, *HBA1* and *HBA2* are the likely candidate genes at this chromosome locus for influencing RBC traits.

We found that a nonsynonymous SNP (rs1050828) in *G6PD*, predicted to be “possibly damaging” by Polyphen2 (Adzhubei *et al.* 2010), was associated with RBC traits. In a candidate gene study of RBC traits in 7112 African Americans, Lo *et al.* (2011) also found rs1050828 in *G6PD* to be associated with HGB (β = −0.241, *P* = 1 × 10^−15^), HCT (β = −0.222, *P* = 1 × 10^−13^), RBC count (β = −0.415, *P* = 4 × 10^−21^), and MCV (β = 0.340, *P* = 3 × 10^−18^). G6PD deficiency is one of the most common enzymopathies in humans (nearly 300 million affected people worldwide), paralleling the distribution of malaria (Beutler 2008; Salvador and Savageau 2003). Previous reports demonstrate that the haplotypic structure at *G6PD* has been shaped by recent positive selection (Tishkoff *et al.* 2001).

In a previous GWAS for RBC traits in a European ancestry cohort of similar sample size (*n* = 3012) (Kullo *et al.* 2010), we identified three loci (*HFE* on chromosome 6p22.2, *HBSL1/MYB* on chromosome 6q23.3, and *TMPRSS6* on chromosome 22q12.3) to be associated with RBC traits. These loci were not associated with RBC traits at a genome-wide significance level in the present study. Only two of the 15 loci that we previously identified in 12,486 patients of European ancestry in the eMERGE network (Ding *et al.* 2012), were replicated in this study. Of additional loci found in other large GWAS (Ganesh *et al.* 2009; Soranzo *et al.* 2009), only variants in *ITFG3* on chromosome 16p13.3 were replicated (*P* < 5 × 10^−8^). One explanation could be that the discovery cohort in the present study is underpowered to detect such associations given the relatively small sample size. The variance explained by 23 loci associated with RBC traits in a prior GWAS (Ganesh *et al.* 2009), ranged from 0.09%~1.12%. In our GWAS for RBC traits in European Americans, variance explained by SNPs at 15 loci ranged from 0.2%~1.4%. For a sample size of 1904 in the discovery cohort, our power to detect such associations is low (10%~40%). Another explanation may be that the genetic architecture underlying RBC traits differs in the two ethnic groups.

Limitations of our study are to be noted. The RBC traits are correlated. We attempted to take this into consideration by performing PCA in which the two PCs that represented majority of the variance in RBC traits were used as phenotypes. The two PCs represented RBC count, HGB, HCT and RBC count, MCV, and MCH, respectively. These analyses demonstrated that the 16p locus was associated with both the PCs whereas the Xq28 was associated with the second PC alone. Our results may have been affected by ascertainment bias, due to different reasons for recruitment at the two sites. Adjustment for site may only partially address this limitation.

Our findings highlight that in individuals of recent African ancestry, variants in three genes known to be protective against malaria are associated with RBC traits. Natural selection increases the frequency of alleles that reduce the deleterious effects in the context of evolution but this could lead to detrimental phenotype effects at the individual level. Thus, protection against malaria comes at the cost of altered RBC traits including lower hemoglobin levels or greater MCV. Our results provide insights into how the powerful force of natural selection might shape variation in medically relevant quantitative traits. Signatures of recent positive selection have also been reported for loci associated with white blood cell traits (Crosslin *et al.* 2012; Nalls *et al.* 2011; Reich *et al.* 2009).

In conclusion, using a GWAS approach, we identified three loci associated with RBC traits in individuals of recent African ancestry. Variants in genes residing in these loci (*i.e.*, *HBB*, *HBA1/HBA2*, and *G6PD*) confer resistance to malaria. None of the three loci identified in a GWAS for RBC traits in a European ancestry cohort of similar sample size (*n* = 3012) (Kullo *et al.* 2010), were associated with RBC traits in African Americans. This may, in part, be due to different genetic architectures underlying RBC traits in the two ethnic groups.

## Supplementary Material

Supporting Information
