# Supplementary material for: Genetic Variants That Confer Resistance to Malaria Are Associated with Red Blood Cell Traits in African-Americans: An Electronic Medical Record-based Genome-Wide Association Study
Source: G3 (Bethesda). 2013 Jul 1;3(7):1061–8. doi: 10.1534/g3.113.006452 (PMC3704235; doi:10.1534/g3.113.006452)
Supplement: Supporting Information [file supp_3_7_1061__index.html]

Genetic Variants That Confer Resistance to Malaria Are Associated with Red Blood Cell Traits in African-Americans: An Electronic Medical Record-based Genome-Wide Association Study — Supporting Information 

# Genetic Variants That Confer Resistance to Malaria Are Associated with Red Blood Cell Traits in African-Americans: An Electronic Medical Record-based Genome-Wide Association Study

## Supporting Information for Ding *et al.*, 2013

**Files in this Data Supplement:**

- Supporting Information - Figures S1-S4 and Tables S1-S2 (PDF, 502 KB)
- Figure S1 - Boxplot of the RBC traits for the genotypes of significant SNPs (PDF, 318 KB)
- Figure S2 - Plot of the first two principal components of the RBC traits (PC1: HCT,HGB, and RBC count; PC2: MCV, MCH, and RBC count). (PDF, 276 KB)
- Figure S3 - Manhattan plot for PC1 (PDF, 278 KB)
- Figure S4 - Manhattan plot for PC2 (PDF, 275 KB)
- Table S1 - The mean ± s.d. values of RBC traits before and after implementing the phenotyping algorithm in the combined cohorts (PDF, 234 KB)
- Table S2 - Pairwise correlation of six RBC traits (PDF, 234 KB)
