# Supplementary material for: Genetic Variants That Confer Resistance to Malaria Are Associated with Red Blood Cell Traits in African-Americans: An Electronic Medical Record-based Genome-Wide Association Study
Source: G3 (Bethesda). 2013 Jul 1;3(7):1061–8. doi: 10.1534/g3.113.006452 (PMC3704235; doi:10.1534/g3.113.006452)
Supplement: Supporting Information [file supp_g3.113.006452_006452SI.pdf]

G3 · Genes | Genomes | Genetics

Genetic Variants that Confer Resistance to Malaria Are Associated with Red Blood Cell Traits in African Americans: An Electronic Medical Record-based Genome Wide Association Study

Keyue Ding,[1] Mariza de Andrade,[2] Teri Manolio,[3] Dana C. Crawford,[4] Laura J. Rasmussen-Torvik,[5] Marylyn D. Ritchie,[6] Joshua C. Denny,[7] Daniel R. Masys,[8] Hayan Jouni,[9] Jennifer A. Pachecho,[10] Abel N. Kho,[10] Dan M. Roden,[7] Rex Chisholm,[11] and Iftikhar J. Kullo[1]

1. Division of Cardiovascular Diseases, Mayo Clinic, Rochester MN 55905, USA
2. Division of Biomedical Statistics and Informatics, Mayo Clinic, Rochester MN 55905, USA
3. Office of Population Genomics, National Human Genome Research Institute (NHGRI), Bethesda, MD 20892, USA
4. Center for Human Genetics Research, Vanderbilt University, Nashville, TN 37232, USA
5. Department of Preventive Medicine, Northwestern University Feinberg School of Medicine, Chicago IL 60611, USA
6. Department of Biochemistry and Molecular Biology, The Pennsylvania State University, University Park, PA 16802, USA
7. Departments of Biomedical Informatics, Medicine and Pharmacology, Vanderbilt University School of Medicine, Nashville, TN 37232, USA
8. Department of Biomedical Informatics and Medical Education, University of Washington, Seattle, WA 98195, USA
9. Department of Internal Medicine, Mayo Clinic, Rochester MN 55905, USA
10. Departments of Medicine and Preventive Medicine, Northwestern University, Chicago, IL 60611, USA
11. Center for Genetic Medicine, Northwestern University Feinberg School of Medicine, Chicago IL 60611, USA

Corresponding author

Iftikhar J. Kullo, M.D.
Mayo Clinic
200 First Street SW
Rochester, MN 55905
Email: Kullo.iftikhar@mayo.edu
Phone: 507-284-9049

DOI: 10.1534/g3.113.006452

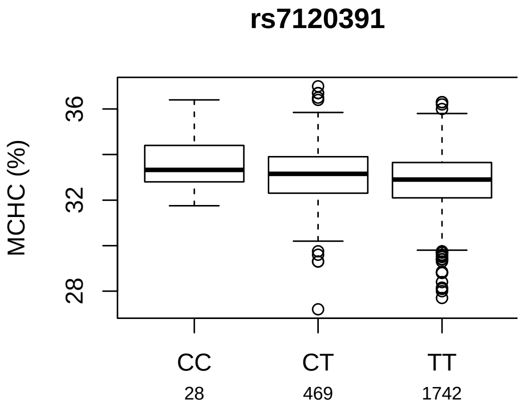

(a)

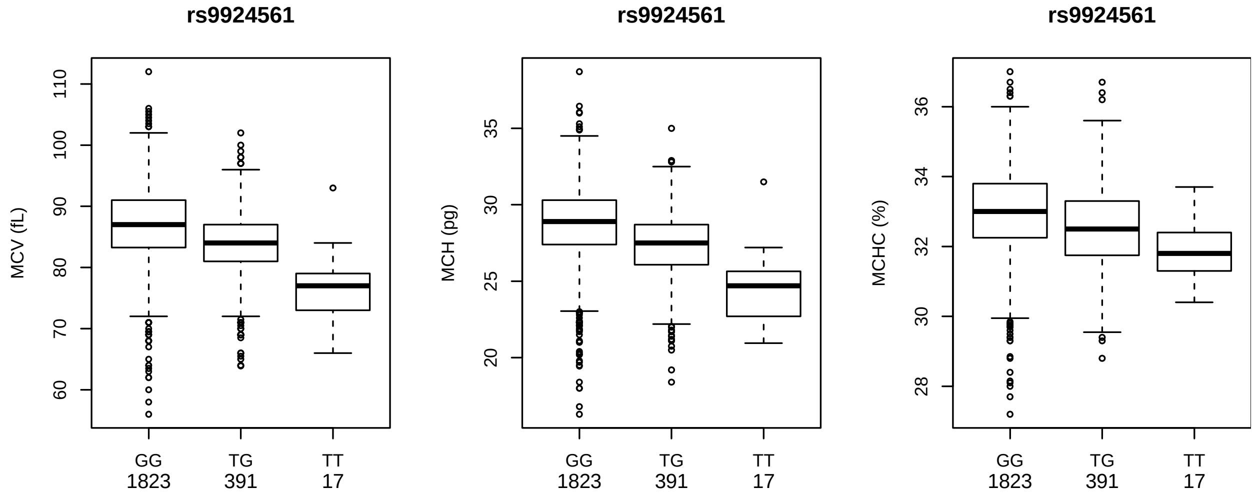

(b)

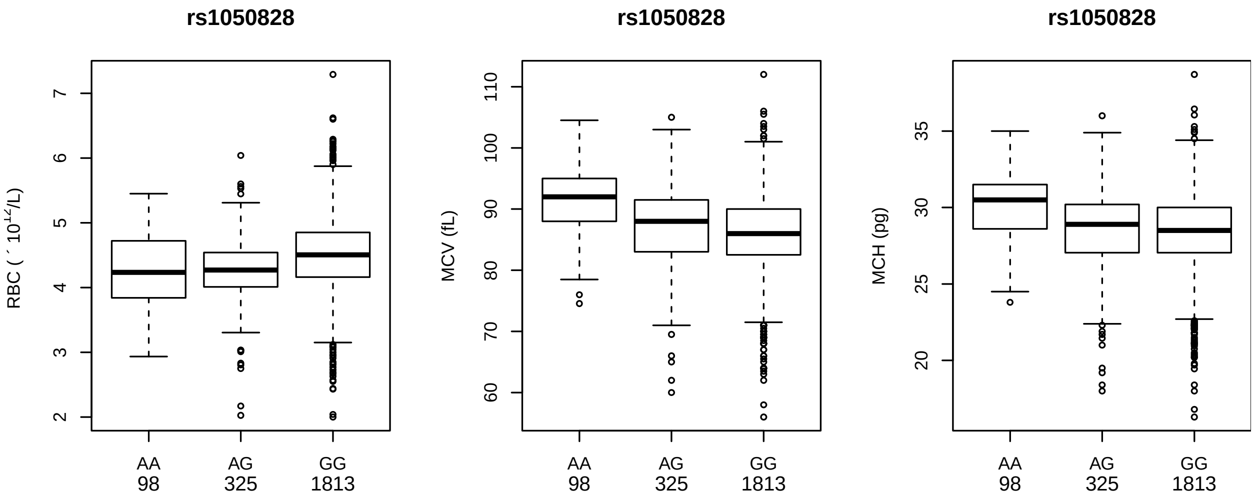

(c)

Figure S1 Boxplot of the RBC traits for the genotypes of significant SNPs. The number of genotype counts was shown below the genotype. The total number of genotypes for the three SNPs are 2239, 2231, and 2236, respectively (instead of 2243 phenotype data shown in Table S2 due to missing genotype data). For rs1050828, genotype 'A' and 'G' in male was included in 'AA' and 'GG'.

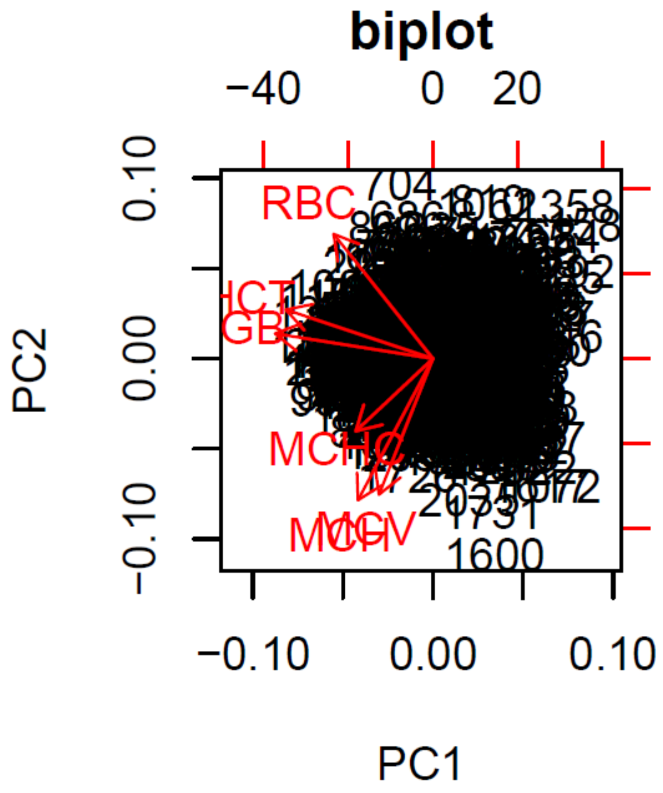

Figure S2 Plot of the first two principal components of the RBC traits (PC1: HCT,HGB, and RBC count; PC2: MCV, MCH, and RBC count).

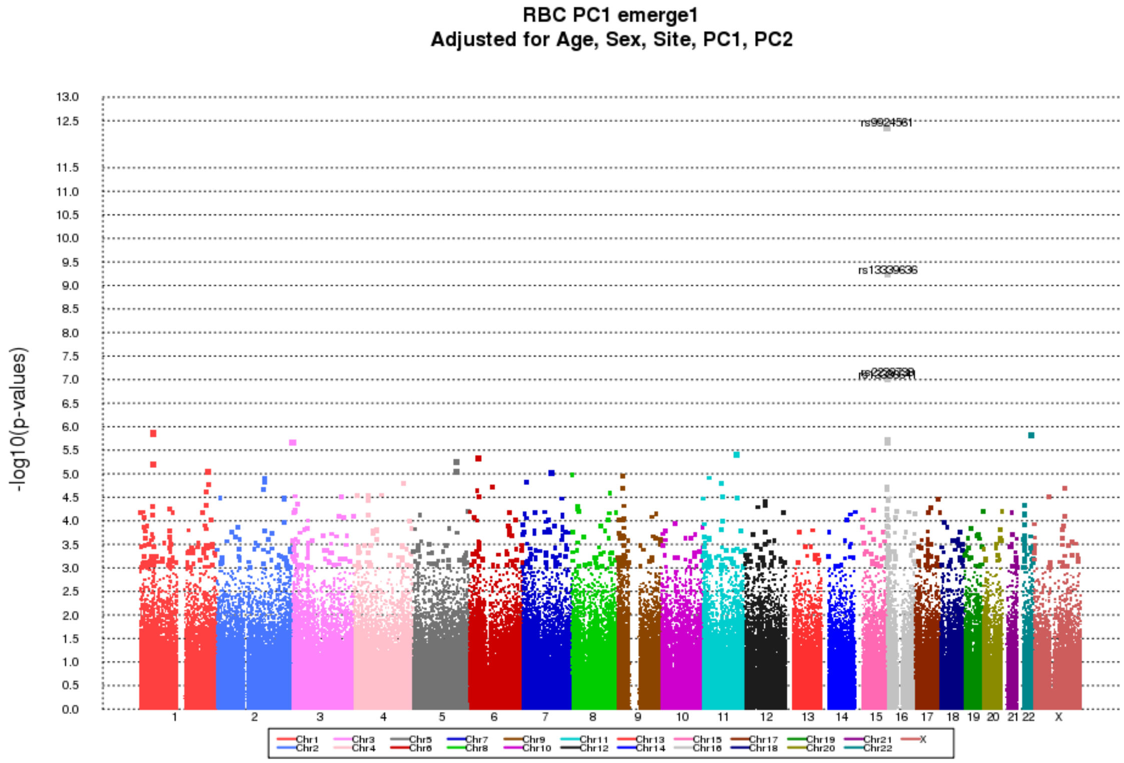

Figure S3 Manhattan plot for PC1

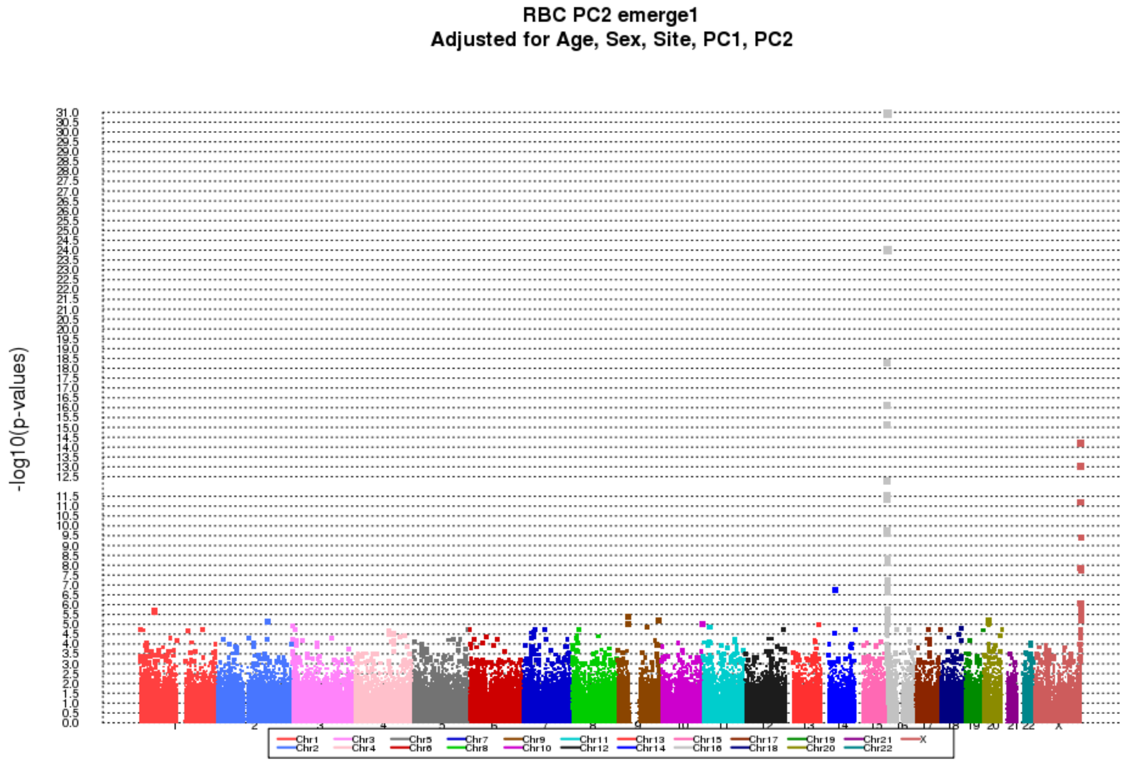

Figure S4 Manhattan plot for PC2

Table S1 The mean ± s.d. values of RBC traits before and after implementing the phenotyping algorithm in the combined cohorts

Trait	Before exclusion	After exclusion[1]	P (Wilcox test)
HGB (g/dL)	12.59±1.58	12.77±1.49	0.0007
HCT (%)	38.26±4.43	38.76±4.12	0.001
RBC count ($\times 10^{12}$/L)	4.45±0.57	4.51±0.52	0.006
MCV (fL)	86.38±6.37	86.41±6.29	0.979
MCH (pg)	28.43±2.5	28.46±2.49	0.715
MCHC (% or g/dL)	32.9±1.19	32.92±1.18	0.401

1. In total, 310 patients were excluded.

Table S2 Pairwise correlation of six RBC traits

	HGB	HCT	RBC count	MCV	MCH	MCHC
HGB	-					
HCT	0.95	-				
RBC count	0.73	0.79	-			
MCV	0.20	0.14	-0.46	-		
MCH	0.32	0.17	-0.39	0.92	-	
MCHC	0.40	0.13	-0.03	0.23	0.59	-
