# Supplementary material for: Genetic Variants That Confer Resistance to Malaria Are Associated with Red Blood Cell Traits in African-Americans: An Electronic Medical Record-based Genome-Wide Association Study
Source: G3 (Bethesda). 2013 Jul 1;3(7):1061–8. doi: 10.1534/g3.113.006452 (PMC3704235; doi:10.1534/g3.113.006452)
Supplement: Supporting Information [file supp_g3.113.006452_FigureS1.pdf]

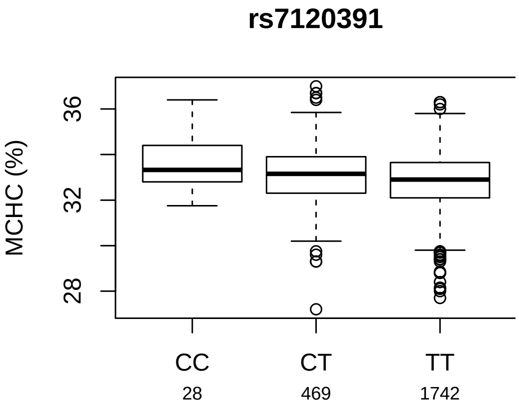

(a)

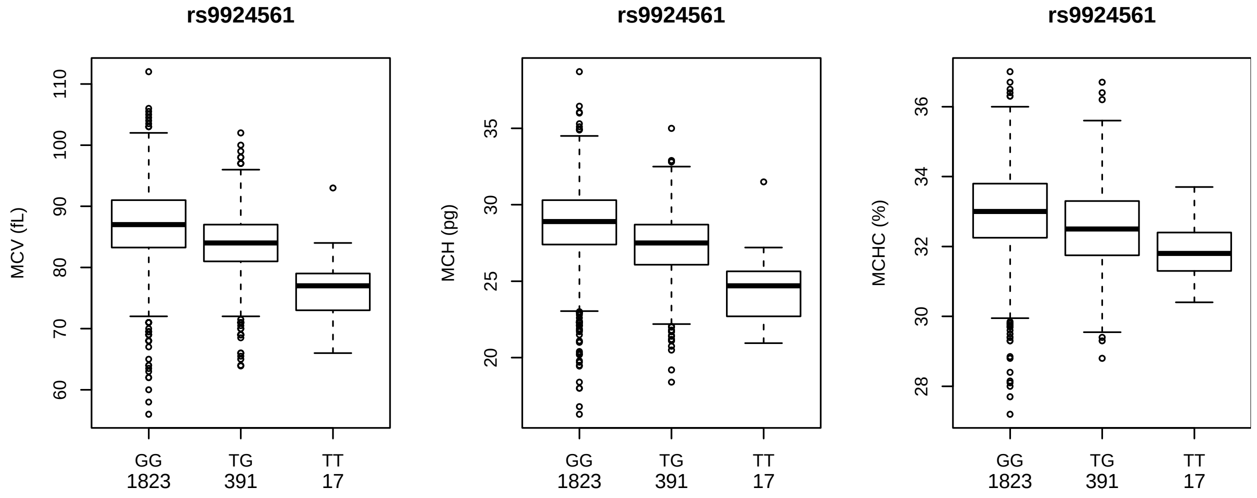

(b)

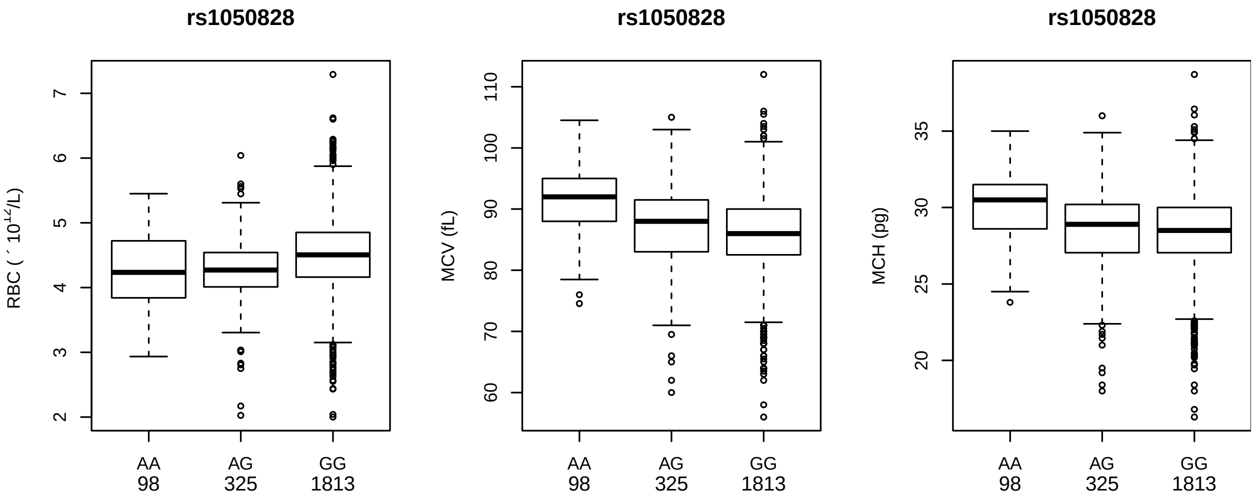

(c)

Figure S1 Boxplot of the RBC traits for the genotypes of significant SNPs. The number of genotype counts was shown below the genotype. The total number of genotypes for the three SNPs are 2239, 2231, and 2236, respectively (instead of 2243 phenotype data shown in Table S2 due to missing genotype data). For rs1050828, genotype ‘A’ and ‘G’ in male was included in ‘AA’ and ‘GG’.
