# Supplementary material for: Genetic Variants That Confer Resistance to Malaria Are Associated with Red Blood Cell Traits in African-Americans: An Electronic Medical Record-based Genome-Wide Association Study
Source: G3 (Bethesda). 2013 Jul 1;3(7):1061–8. doi: 10.1534/g3.113.006452 (PMC3704235; doi:10.1534/g3.113.006452)
Supplement: Supporting Information [file supp_g3.113.006452_FigureS2.pdf]

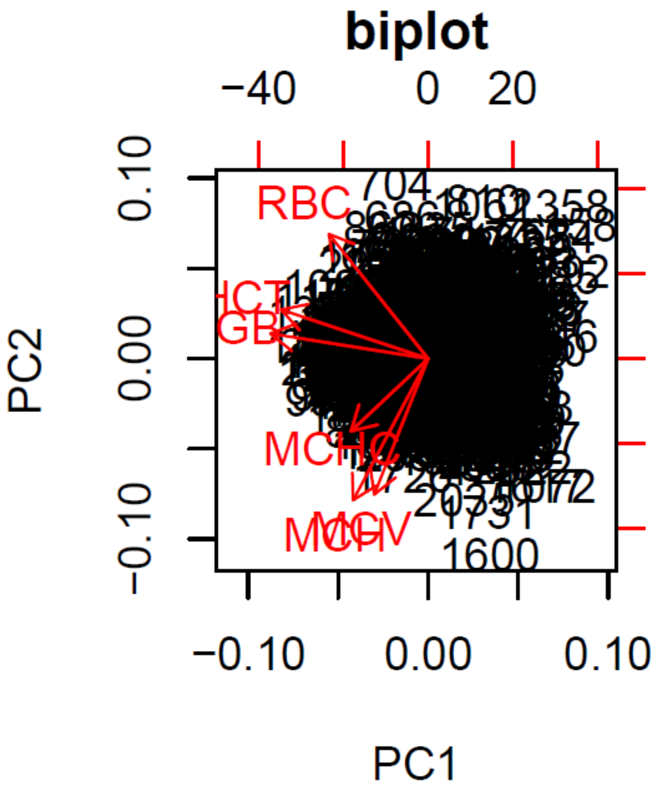

Figure S2 Plot of the first two principal components of the RBC traits (PC1: HCT,HGB, and RBC count; PC2: MCV, MCH, and RBC count).
