# Supplementary figures and images for: Genetic Variants That Confer Resistance to Malaria Are Associated with Red Blood Cell Traits in African-Americans: An Electronic Medical Record-based Genome-Wide Association Study
Source: G3 (Bethesda). 2013 Jul 1;3(7):1061–8. doi: 10.1534/g3.113.006452 (PMC3704235; doi:10.1534/g3.113.006452)

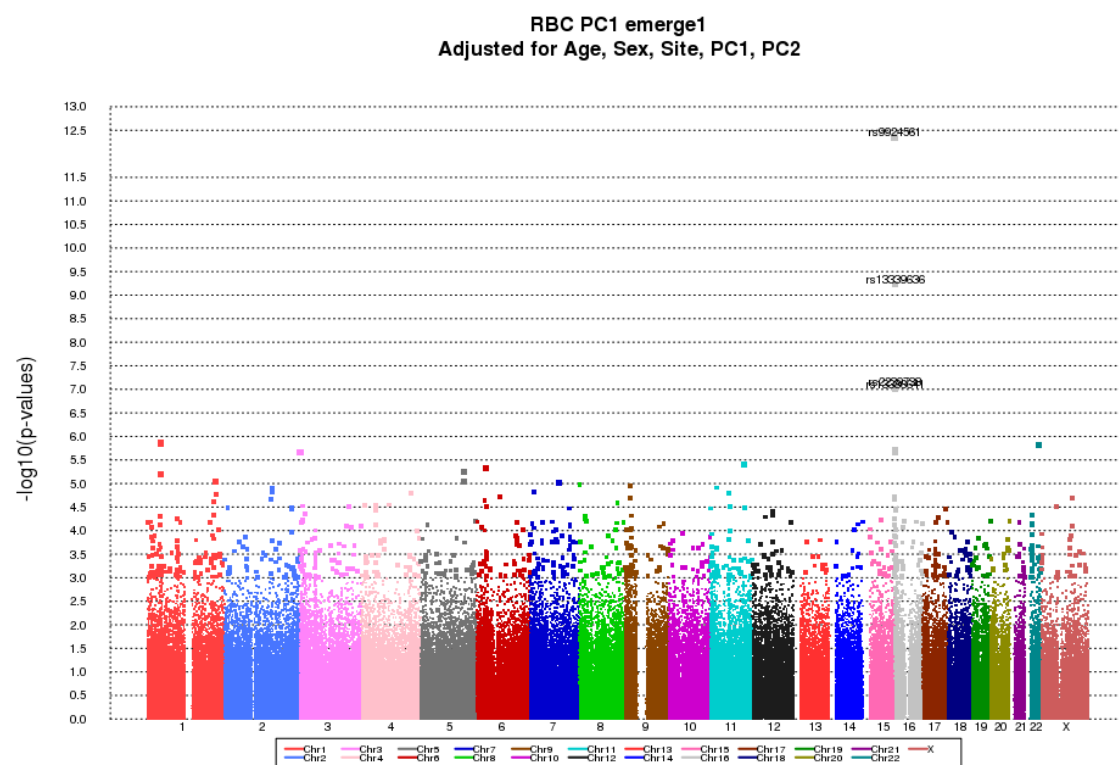

**Figure S3** Manhattan plot for PC1

Supplement: Supporting Information [file supp_g3.113.006452_FigureS3.pdf]

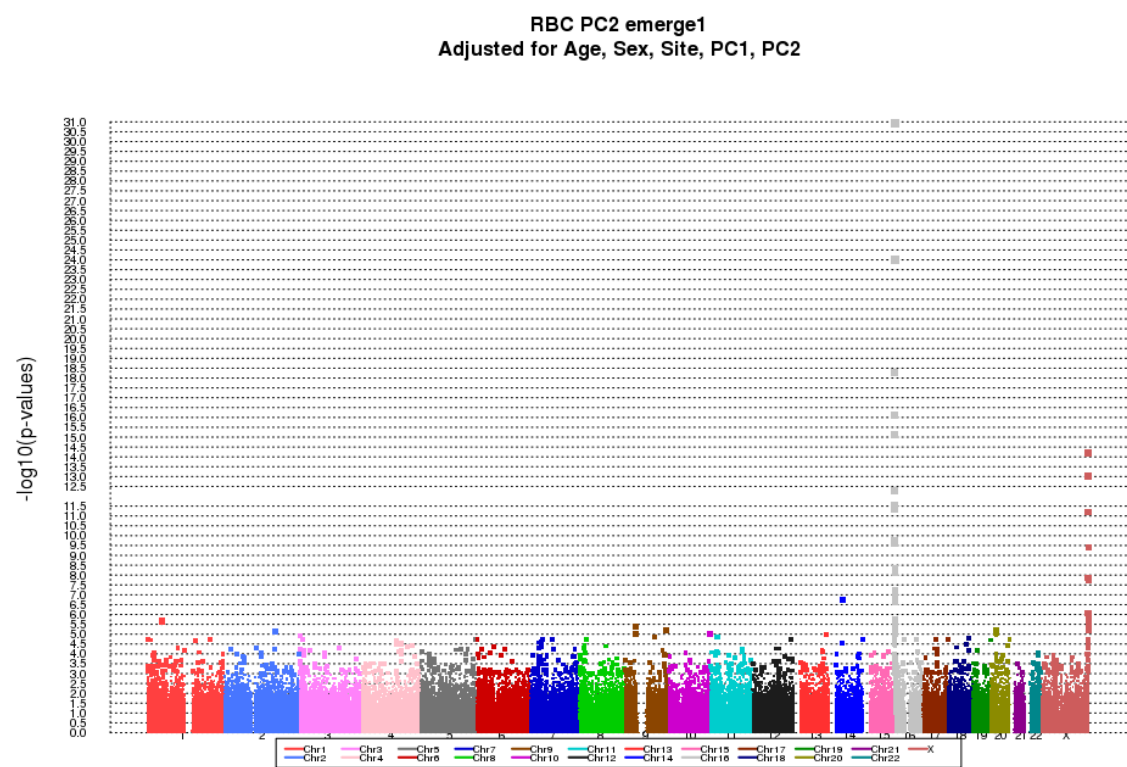

**Figure S4** Manhattan plot for PC2

Supplement: Supporting Information [file supp_g3.113.006452_FigureS4.pdf]
