# Supplementary material for: Genetic Variants That Confer Resistance to Malaria Are Associated with Red Blood Cell Traits in African-Americans: An Electronic Medical Record-based Genome-Wide Association Study
Source: G3 (Bethesda). 2013 Jul 1;3(7):1061–8. doi: 10.1534/g3.113.006452 (PMC3704235; doi:10.1534/g3.113.006452)
Supplement: Supporting Information [file supp_g3.113.006452_TableS1.pdf]

Table S1 The mean ± s.d. values of RBC traits before and after implementing the phenotyping algorithm in the combined cohorts

Trait	Before exclusion	After exclusion[1]	P (Wilcox test)
HGB (g/dL)	12.59±1.58	12.77±1.49	0.0007
HCT (%)	38.26±4.43	38.76±4.12	0.001
RBC count ($\times 10^{12}$/L)	4.45±0.57	4.51±0.52	0.006
MCV (fL)	86.38±6.37	86.41±6.29	0.979
MCH (pg)	28.43±2.5	28.46±2.49	0.715
MCHC (% or g/dL)	32.9±1.19	32.92±1.18	0.401

1. In total, 310 patients were excluded.
