# Supplementary material for: Genetic Variants That Confer Resistance to Malaria Are Associated with Red Blood Cell Traits in African-Americans: An Electronic Medical Record-based Genome-Wide Association Study
Source: G3 (Bethesda). 2013 Jul 1;3(7):1061–8. doi: 10.1534/g3.113.006452 (PMC3704235; doi:10.1534/g3.113.006452)
Supplement: Supporting Information [file supp_g3.113.006452_TableS2.pdf]

Table S2 Pairwise correlation of six RBC traits

	HGB	HCT	RBC count	MCV	MCH	MCHC
HGB	-					
HCT	0.95	-				
RBC count	0.73	0.79	-			
MCV	0.20	0.14	-0.46	-		
MCH	0.32	0.17	-0.39	0.92	-	
MCHC	0.40	0.13	-0.03	0.23	0.59	-
